# First Laboratory-Confirmed Outbreak of Human and Animal Rift Valley Fever Virus in Uganda in 48 Years

**DOI:** 10.4269/ajtmh.18-0732

**Published:** 2019-01-21

**Authors:** Trevor R. Shoemaker, Luke Nyakarahuka, Stephen Balinandi, Joseph Ojwang, Alex Tumusiime, Sophia Mulei, Jackson Kyondo, Bernard Lubwama, Musa Sekamatte, Annemarion Namutebi, Patrick Tusiime, Fred Monje, Martin Mayanja, Steven Ssendagire, Melissa Dahlke, Simon Kyazze, Milton Wetaka, Issa Makumbi, Jeff Borchert, Sara Zufan, Ketan Patel, Shannon Whitmer, Shelley Brown, William G. Davis, John D. Klena, Stuart T. Nichol, Pierre E. Rollin, Julius Lutwama

**Affiliations:** 1 Viral Special Pathogens Branch, Centers for Disease Control and Prevention-Uganda, Entebbe, Uganda;; 2 Viral Special Pathogens Branch, Centers for Disease Control and Prevention, Atlanta, Georgia;; 3 Department of Arbovirology, Emerging and Reemerging Infectious Diseases, Uganda Virus Research Institute, Entebbe, Uganda;; 4 Department of Biosecurity, Ecosystems and Veterinary Public Health, College of Veterinary Medicine, Animal Resources and Biosecurity, Makerere University, Kampala, Uganda;; 5 Global Health Security Unit, Centers for Disease Control and Prevention-Uganda, Kampala, Uganda;; 6 Ministry of Health, Kampala, Uganda;; 7 Kabale Regional Referral Hospital, Kabale, Uganda;; 8 Kabale District Health Office, Kabale, Uganda;; 9 Ministry of Agriculture, Animal Industry and Fisheries, Kampala, Uganda;; 10 Public Health Emergency Operations Centre, Ministry of Health, Kampala, Uganda

## Abstract

In March 2016, an outbreak of Rift Valley fever (RVF) was identified in Kabale district, southwestern Uganda. A comprehensive outbreak investigation was initiated, including human, livestock, and mosquito vector investigations. Overall, four cases of acute, nonfatal human disease were identified, three by RVF virus (RVFV) reverse transcriptase polymerase chain reaction (RT-PCR), and one by IgM and IgG serology. Investigations of cattle, sheep, and goat samples from homes and villages of confirmed and probable RVF cases and the Kabale central abattoir found that eight of 83 (10%) animals were positive for RVFV by IgG serology; one goat from the home of a confirmed case tested positive by RT-PCR. Whole genome sequencing from three clinical specimens was performed and phylogenetic analysis inferred the relatedness of 2016 RVFV with the 2006–2007 Kenya-2 clade, suggesting previous introduction of RVFV into southwestern Uganda. An entomological survey identified three of 298 pools (1%) of *Aedes* and *Coquillettidia* species that were RVFV positive by RT-PCR. This was the first identification of RVFV in Uganda in 48 years and the 10^th^ independent viral hemorrhagic fever outbreak to be confirmed in Uganda since 2010.

## INTRODUCTION

Rift Valley fever (RVF) is caused by RVF virus (RVFV), a single-stranded RNA virus in the *Bunyavirales* order, *Phenuiviridae* family, and *Phlebovirus* genus. It is an emerging epidemic disease of humans and livestock and an important endemic problem in sub-Saharan Africa.^1^ Rift Valley fever virus is transmitted to livestock and humans by the bite of infected mosquitoes or exposure to tissues or blood of infected animals.^2^ Interepidemic virus maintenance is thought to occur either transovarially in *Aedes* species mosquitoes or through cycling of low-level transmission between domestic livestock or wild ungulates and mosquitoes.^3^ After periods of heavy rainfall, *Aedes* mosquitoes rapidly emerge, resulting in extensive amplification of the virus through infection of livestock.^2^

Presentation of RVF in animals can vary among species with a range of clinical severity. Livestock, particularly cattle, sheep, and goats are highly susceptible to RVFV and present with symptoms of fever, loss of appetite, weakness, low milk production, nasal discharge, vomiting, and diarrhea. During large epizootics, “abortion storms,” particularly in sheep and cattle, have been identified. High newborn mortality (80–100%) and adult mortality (5–20%) may also be observed.^3^

Humans infected with RVFV typically have mild, self-limited febrile illness, but can present in a small number of cases (< 8%) with severe jaundice, rhinitis, encephalitis, and hemorrhagic manifestations. Retinal degeneration (5–10% of cases), hemorrhagic fever (< 1%), or encephalitis (< 1%) may also develop.^4,5^ Outbreaks of RVF have been reported most frequently in East Africa, especially Kenya, Somalia, and Tanzania, with the last major outbreak in the region recorded in 1997–1998. However, outbreaks have also been reported in other African countries, including Egypt, South Africa, Madagascar, and Senegal.^6^ In 2000, the first RVF outbreak outside of Africa was reported in Saudi Arabia and Yemen.^5,7^ More recently, Kenya and Tanzania experienced a large epizootic of RVFV in 2006–2007 and Sudan in 2010.^6^ The emergence of RVFV in East Africa resulted in widespread livestock morbidity and mortality, with hundreds of laboratory-confirmed human cases, and likely thousands of asymptomatic or mild RVFV human infections going undetected.^8,9^

Rift Valley fever virus in Uganda was first detected in mosquitos collected in Semliki forest, Western Uganda, in 1944,^10^ and has since been detected several times by the East African Virus Research Institute (EAVRI), now the Uganda Virus Research Institute (UVRI). Human cases were recorded during outbreaks occurring near Entebbe in 1960 and 1962.^11,12^ Since then, serological evidence of human and livestock RVFV infections in Uganda have been intermittently reported,^13,14^ but the last published description of acute human RVFV infection was seven cases occurring near Entebbe in 1968.^15^

On March 10, 2016, UVRI and the Uganda Ministry of Health (MOH) received a report of a suspected viral hemorrhagic fever (VHF) case presenting to Kabale Regional Referral Hospital (KRRH) in Kabale district in southwestern Uganda. The initial case was of a 48-year-old male butcher who had been working in a local abattoir, where livestock were brought for slaughter. The patient presented with a history of fever, vomiting, diarrhea, headache, and hemorrhagic symptoms (bleeding gums, epistaxis, bloody urine, and stools). A blood sample was taken and sent to the UVRI VHF laboratory for testing. On March 11, a second suspected VHF case presented to KRRH with similar symptoms; a blood sample was collected for testing. This patient was a 16-year-old male student first reported by Kabale District Health Office from the Uganda–Rwanda border village of Katuna. Both samples were tested for hemorrhagic fever viruses, including Ebola viruses, Marburg virus, Crimean–Congo hemorrhagic fever virus, and RVFV. Both specimens were found to be positive for RVFV by reverse transcriptase polymerase chain reaction (RT-PCR) and IgM serology.

## MATERIALS AND METHODS

### Outbreak investigation.

Following laboratory confirmation of the initial two cases, a multidisciplinary team from the Uganda MOH, UVRI, the Ministry of Agriculture, Animal Industry and Fisheries (MAAIF), Centers for Disease Control and Prevention (CDC)-Uganda, World Health Organization (WHO), and Kabale district local government performed the initial investigation. The primary objectives were to perform a detailed epidemiological case investigation, initiate enhanced surveillance and active case finding, establish a designated treatment unit at KRRH, and survey active animal morbidity and mortality. The epidemiology and surveillance team provided case definitions (suspected, probable, and confirmed) for RVF and helped identify new human cases in the community. A suspected case was anyone presenting with acute onset of fever (> 37.5°C), a negative malaria test, and at least two of the following three symptoms: headache, muscle or joint pain, and any gastroenteritis symptom (nausea, vomiting, abdominal pain, and diarrhea). A probable case was any unexplained death in the community, and any suspected case that had thrombocytopenia, low white blood cell counts, or raised hematocrit, plus at least one of the following: bleeding tendencies, sudden change in vision, or jaundice. A confirmed case was any suspected or probable case with a laboratory confirmation by either detection of RVFV nucleic acid by RT-PCR or demonstration of serum IgM antibodies by ELISA.

Within Kabale district, specialized teams were formed to investigate newly suspected RVF cases and respond to reported animal morbidity or mortality. Teams conducted a detailed clinical description, established the clinical status of listed cases, and verified new and prior suspected cases in the community and health facilities. The standard VHF case report form was used for collecting data of clinical signs and symptoms and epidemiological risk factors, and these were used for hypothesis generation and outbreak case definition updates. Investigation teams also trained local medical and veterinary staff on safe specimen collection methods and provided appropriate sample collection materials and data collection tools.

The laboratory team worked to improve rapid case detection by facilitating sample collection and transport, and fast-tracking RVFV testing of field and hospital specimens taken from humans and livestock. Training field- and hospital-based personnel in the proper use of personal protective equipment while collecting, processing, and handling specimens was also conducted. Additional investigation teams visited high-risk locations to identify further RVF cases; these locations included the main abattoirs, district health centers, and hospitals, where human, livestock, and vector samples were obtained from.

### Investigation of confirmed and probable cases.

The team visited the homes of the two confirmed acute case patients (AC1 and AC2) to collect additional information on the cases and assess potential exposure and risk factors. The team interviewed family members and community residents in attempts to identify additional cases. Investigations were focused around the households of the confirmed patients and extended to the neighboring villages and Kabale town center where the patients worked and were potentially exposed. Additional villages within Kabale district were investigated based on the information gathered during the interviews with the confirmed patients and their family members. Interviews using standard case investigation forms were conducted with all identified persons. A proxy relative or an immediate family member was interviewed if the infected person had died. These interviews extended to other members of the household. Blood samples were collected from all suspected patients and from family members with whom an interview had been conducted.

In addition to the two acute cases, AC1 and AC2, a suspected probable RVF death, probable case 1 (PC1), was reported to the district surveillance officer in Burorane village, Kyanamira subcounty, on March 9. On March 11, one additional report was received of a death suspected to be caused by RVFV in Mushenyi village, Katuna, near the Rwandan border, and listed as probable case 2 (PC2). Investigation teams were dispatched to obtain more detailed information and collect blood samples from close family members and domestic livestock from near the households of PC1 and PC2.

### Community investigations.

**Figure 1.**
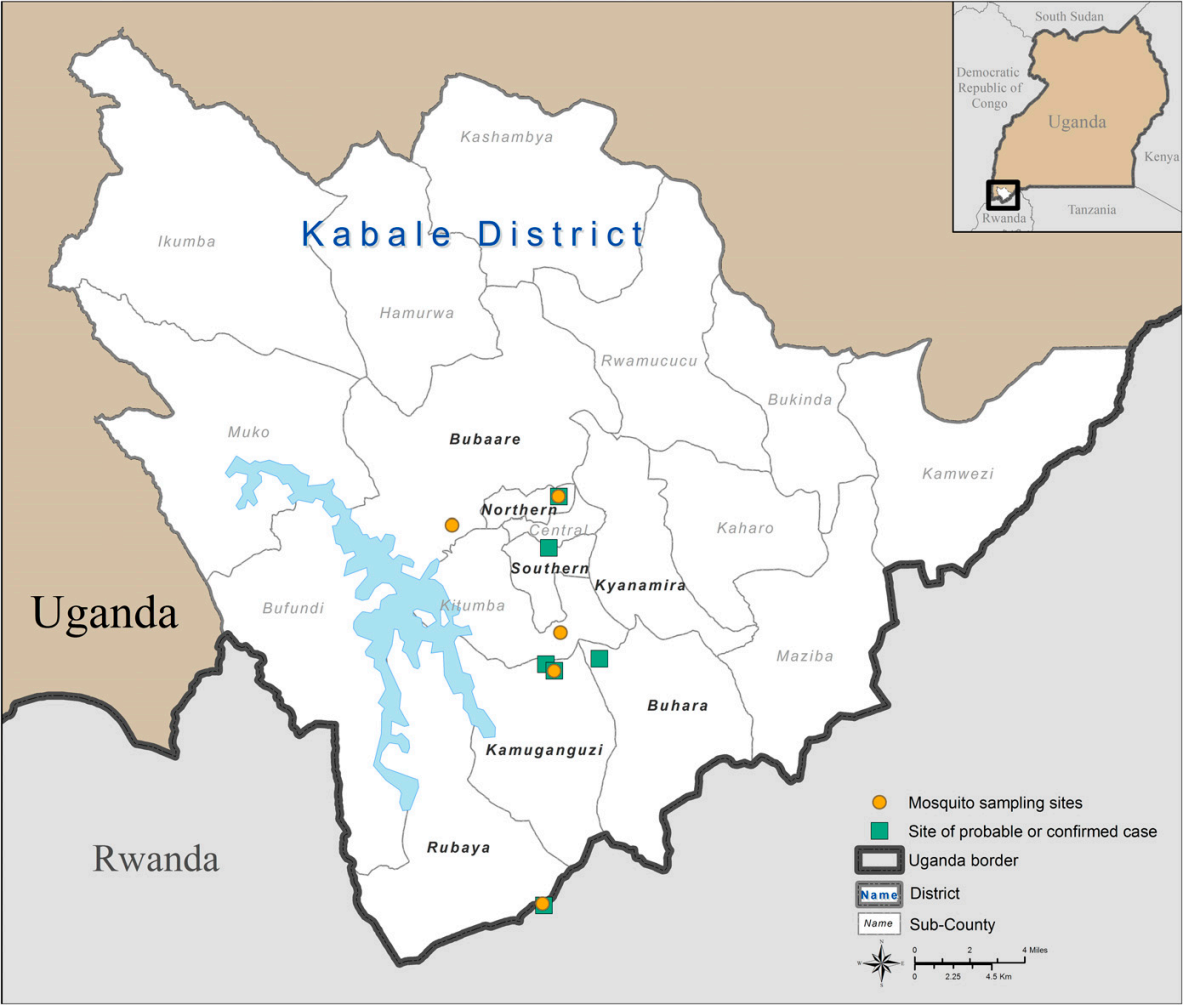
Map showing the locations of confirmed and probable Rift Valley fever cases and locations where human, livestock, and mosquito samples were collected during the outbreak investigations in Kabale district, Uganda, 2016. This figure appears in color at www.ajtmh.org.

The investigation team collected blood samples from household members of confirmed and probable patients, as well as from neighbors in the affected villages, to identify any additional acute or convalescent RVFV cases. In total, 19 human blood samples were collected during the initial rapid investigation from various locations in Kabale district (Figure 1). All samples were tested for RVFV by RT-PCR and for anti-RVFV IgM and IgG by ELISA.

### Livestock investigations.

In addition to collecting human blood samples during the initial investigation, we collected blood samples from domestic livestock in the same locations. Because RVF in animals may be clinically hard to define, blood samples were collected at or near the households where suspected, probable, or confirmed human cases had been identified (Figure 1). Blood samples were also taken from livestock at the central Kabale town abattoir. In total, 83 livestock samples were obtained from bovine, caprine, and ovine species. There were anecdotal reports of increased numbers of sick animals and abortions in the areas near the villages of probable and confirmed cases. Whole blood samples were collected from the jugular veins using vacutainer ethylenediaminetetraacetic acid (EDTA) collection tubes. Information about the clinical history of individual animals and the herd was also recorded. The samples were tested at the UVRI VHF laboratory by both RT-PCR and IgG serology.

### Entomological investigations.

Mosquitoes were collected in five locations in Kabale district (Figure 1). Mosquitoes were trapped in Bugongi village in Northern division near the home of first confirmed case and in Omururinda village, Kamuganguzi subcounty, near the home of the second confirmed case. They were also collected in Mushenyi village, Rubaya subcounty; in the home of a probable case in Kazigizigi village, Kitumba subcounty, located near Kabale town council; and in Nyakayenje village in Bubaale subcounty near Lake Bunyoni (Figure 1). Collections were carried out from March 14 to March 17, 2016, for 2 days in each of the first two villages and then for 1 day on March 23 through 26 in the other three villages. A total of 40 CDC light traps per collection site were baited with dry ice (CO_2_ source), set around homesteads and in shrubs and forested areas near homes. The light traps were set about 50 m from each other in the evening and removed the following morning.

Mosquitoes were then stored at −80°C until identification and processing for virus isolation. Mosquitoes were identified using appropriate keys,^16–18^ processed for identification on a chill table mounted on a Zeiss Discovery V12 microscope (Zeiss, Thornwood, NY), and pooled according to village, gender, and feeding status. Virus isolations were attempted in Vero-E6 cells as previously described.^19–21^

### RNA extraction.

We used the MagMAX magnetic bead system (Life Technologies, Carlsbad, CA) according to the manufacturer’s protocol to extract RNA from all human and livestock samples. Briefly, 100 µL of each specimen was mixed with 400 µL of lysis buffer supplemented with 2 µL of carrier RNA solution. RNA was extracted using a BeadRetriever automated magnetic bead separation system, and then eluted in 90 µL of elution buffer (Applied Biosystems, Inc., Waltham, MA).

### Reverse transcriptase polymerase chain reaction.

The RVF RT-PCR assay used during this investigation has been previously described.^22^ Briefly, the assay uses the following primer and probe set: 5′-TGAAAATTCCTGAGACACATGG-3′ (RVFL-2912fwdGG), 5′-ACTTCCTTGCATCATCTGATG-3′ (RVFL-2981revAC), and FAM-5′-CAATGTAAGGGGCCTGTGTGGACTTGTG-3′-BHQ (RVFL-probe-2950). The samples were cycled on an ABI Quant Studio 5 (ABI) under the following conditions: one cycle of 51°C for 30 minutes and 94°C for 2 minutes, followed by 40 cycles of 94°C for 15 seconds, 56°C for 30 seconds, and 68°C for 2 minutes, followed by a final extension at 68°C for 5 minutes.

### IgM and IgG serology.

Serology was performed on both human and livestock specimens using procedures previously described.^23,24^ One ∼4-mL blood sample was collected from each human participant for serological testing at UVRI by anti-RVFV IgM and IgG ELISA. An ∼4-mL blood sample was collected from each animal for serological testing for RVFV IgG by ELISA. Briefly, whole blood specimens were centrifuged to obtain serum samples. Following heat and detergent inactivation, the specimens were tested by anti-RVFV–specific IgM and IgG ELISA using inactivated RVFV-infected Vero-E6 cell antigens. Four dilutions of each specimen were tested: 1:100, 1:400, 1:1,600, and ≥ 1:6,400. Titers and the cumulative sum optical densities of each dilution (SUMOD) minus the background absorbance of uninfected control antigen (adjusted SUMOD) were recorded. Samples were deemed positive if both the adjusted SUMOD and titer were above pre-established conservative cutoff values of ≥ 0.45 for IgM ELISA and ≥ 0.95 for IgG ELISA. Human samples that were IgM positive were subsequently tested by RT-PCR.

### Virus isolation.

Vero-E6 monolayers in 25 cm^2^ flasks were inoculated with 100 µL of blood, and the cells were incubated at 37°C in 2% fetal bovine serum minimum essential media (FBS MEM) for 14 days, with a media change on day 7. Flasks were checked daily for cytopathic effect, and cells scraped on days 7 and 14 were examined by immunofluorescence. Only the initial blood samples drawn from the three acute RVFV patients yielded RVFV isolates in culture, but no isolates were obtained from any of the subsequent blood draw samples.

### Genetic sequencing and phylogenetic analysis.

RNA was extracted directly from clinical samples using MagMAX Pathogen RNA/DNA isolation kit (Thermo Fisher, Waltham, MA). Libraries for next-generation sequencing were prepared using the NEBNext Ultra RNA kit (New England Biolabs, Ipswich, MA) and sequenced on an Illumina MiSeq (Illumina, San Diego, CA) using version 2 2 × 150 cycle kits. Genomes were assembled using Geneious v9.1.2. (Geneious, Newark, NJ) and viral-ngs with a custom RVFV database.^25^ The evolutionary history was inferred using all available full-length RVFV genomes from GenBank using phyml (GTR + Γ [*n* = 4] –s SPR) with bootstrap support provided by 1,000 iterations. Rift Valley fever virus genomes were deposited to GenBank: MG9534218-26.

### Data management and analysis.

An outbreak database was created using the EpiInfo VHF application. Data from human and livestock investigations were collected using the standard VHF case reporting form and livestock data assessment form and then entered at the district headquarters into the EpiInfo VHF application. Periodic extracts were electronically sent to Kabale district surveillance officers and to the MOH Public Health Operations Center in Kampala for summary data and generation of national situation reports. Human data analysis was performed within the EpiInfo VHF application using EpiInfo 7 (Centers for Disease Control and Prevention, Atlanta, GA).

### Ethical considerations.

Approval for outbreak investigations was authorized through the established procedures of the Uganda MOH National Task Force for Epidemics, which was activated and met daily at the Public Health Operations Center in Kampala. In addition, ethical approval from UVRI and CDC was obtained through a determination that the investigations were part of an outbreak; the investigations were classified as non-research. Participants provided oral consent before completing case report forms and interviews. Participants provided consent to collect a blood sample from themselves and from their animals, if applicable. In addition, participants were informed that blood samples may be tested for other zoonotic pathogens at UVRI as part of the ongoing investigation. Parents of minors included in the investigations consented to minors’ participation before interviewing. All participation was voluntary, and participants did not receive any compensation.

## RESULTS

### Confirmed case investigations.

The initial acute case, AC1, was a 48-year-old male butcher from Upper Bugongi, Northern division, Kabale municipality. On March 2, 2016, he reported an acute onset of a high-grade fever, severe headache, and joint pains. On March 3, he traveled to KRRH to seek treatment for suspected malaria, was discharged, and returned home. By March 9, 7 days after onset of symptoms, he still had a fever and had developed other symptoms that included loss of appetite, generalized body weakness, severe anemia, ocular jaundice, and hemorrhagic symptoms (bleeding from the conjunctiva and nose, and bloody stool). He reported back to KRRH and was admitted for high risk of renal and liver failure. The attending physicians suspected VHF and obtained a blood sample to send to UVRI for testing. The sample was received at the UVRI VHF laboratory on March 10, tested, and found positive for RVFV by RT-PCR (cycle threshold [Ct] = 32).

The second acute case, AC2, was a 16-year-old male student from Omururinda village, Kitumba parish, Kamuganguzi subcounty. Records at KRRH showed that he first reported to the outpatient facility on February 10, 2016, with a 10-day history of fever, headaches, and joint pain. On this visit, he tested negative for malaria by both microscopy and malaria rapid diagnostic test (mRDT). He was treated with cotrimoxazole (Septrin) and mebendazole. On February 22, he presented to Kamukira Health Center IV in Kabale town reporting symptoms consistent with relapsing fever. He again sought care to manage the same fever as an outpatient at KRRH on February 25 and was given antimalarial drugs, treated symptomatically, and discharged. On March 10, he presented at KRRH again with a high-grade fever (39°C) and hemorrhagic signs, including bloody sputum, nose bleeding, bloody urine, and bloody stool for 3 days, as well as a very tender abdomen. He had low platelet counts and was anemic (hemoglobin 7.9 g/dL) with high amylase levels but normal creatinine and was treated for epistaxis with cotton nasal packs. He had tested negative for malaria by microscopy, for typhoid fever and hepatitis B by HbSAg, and for brucellosis by the BAT (buffered Brucella antigen test). A blood sample was collected and sent to UVRI for testing suspected VHF. He was isolated the following day and given supportive therapy. By the time of his isolation, he had started convulsing and remained unconscious for 6 days. On March 11, RVFV was detected in the sample by RT-PCR (Ct = 30).

### Probable case investigations.

Probable case 1 (PC1) was a 30-year-old male butcher from Burorane village, Kyanamira subcounty, Kabale district, who died on February 16, 2016. The investigation team interviewed family members, who reported that his symptoms began on February 13 and included headache, fever, and diarrhea, followed by convulsions and mental disorientation. On February 14, he developed hemorrhagic signs, including nose bleed and vomiting blood, as well as hiccups and loss of consciousness. Bleeding was reported to continue following his death. One of his sisters reportedly fell ill earlier in the month and had recovered. The team collected two blood samples from the patient’s wife and a brother who cared for him during his illness and from two additional family members. No RVFV infection was detected in these persons by IgM or IgG serology. No blood samples were collected from domestic livestock at this homestead (Table 1).

**Table 1:** Summary of human and livestock sampling and testing, Kabale district, 2016

Investigation site (subcounty)	RVFV IgM seropositive	RVFV IgG seropositive	RT-PCR positive	Note
Central division				
Household #1				
Human (*n* = 0)	–	–	–	Home of case AC1
Livestock (*n* = 5)	–	0 (0%)	0 (0%)	
Abattoir				
Livestock (*n* = 45)	–	2 (4.4%)	0 (0%)	Kabale central abattoir
Kamuganguzi				
Human (*n* = 5)	0 (0%)	0 (0%)	–	Home of AC2
Livestock (*n* = 12)	–	2 (16.7%)	1 (8.3%)	
Kyanamira				
Human (*n* = 4)	0 (0%)	0 (0%)	–	Home of PC1
Livestock (*n* = 0)	–	–	–	
Rubya				
Household #1				
Human (*n* = 5)	1 (20%)	2 (40%)	–	
Livestock (*n* = 10)	–	4 (40%)	0 (0%)	Home of PC2
Household #2				
Human (*n* = 4)	0 (0%)	0 (0%)	–	Home of grandparents of AC2
Livestock (*n* = 11)	–	0 (0%)	0 (0%)	

RT-PCR = reverse transcriptase polymerase chain reaction; RVFV = Rift Valley fever virus.

Probable RVF case 2 (PC2) was a 30-year-old male farmer from Mushenyi village, Katuna, near the Rwandan border, who first felt ill on March 4. He resided less than a kilometer from the residence of the grandparents of AC2 but was not otherwise epidemiologically linked to the grandparents of AC2. This patient experienced symptoms of headache, fever, muscle pains, bleeding from the mouth, mental disorientation, and loss of vision and was taken to a clinic near the border, where he received IV fluids. He was later transferred to the Rugarama hospital near Kabale town, where he died on March 10 and was buried on March 12. Blood samples from five family members and 10 domestic livestock were collected at the homestead and tested. No close family members showed evidence of RVFV infection by IgM or IgG serology (Table 1). IgG serology detected RVFV in four livestock samples.

### Community investigations.

No additional acute human RVFV cases were identified by RT-PCR. Low levels of both IgM and IgG were detected in one sample collected from a 36-year-old male farmer residing in Mushenyi village, Rubaya subcounty, approximately 25 km south of Kabale town (Table 1). He had no epidemiological linkages to AC1, AC2, PC1, or PC2. Mushenyi village is approximately 3 km from Omururinda village where the second confirmed case resided and was presumed to have been infected. The man reported onset of fever on March 3 but began to recover starting on March 10. Other symptoms included intense fatigue, chest and muscle pains, and headache. On March 13, a sample was collected from him during the initial outbreak investigation and sent to UVRI VHF laboratory. No RVFV was detectable by RT-PCR, and anti-RVFV IgM and IgG titers were 1:400. This case was classified as the third acute confirmed case, AC3.

In addition, one convalescent IgG-seropositive case was identified, convalescent case 1 (CC1), from Mushenyi village in a 26-year-old male farmer. He reported some symptoms compatible with RVF, such as fever, headache, abdominal pain, fatigue, and chest pain, but did not seek medical care. Although he had contact with livestock, none of the animals were sick recently or at the time of sampling.

A second convalescent IgG-seropositive case was identified, convalescent case 2 (CC2), from Burorane village, Kyanamira subcounty. The patient was a carpenter and the brother of the second probable death described previously. At the time of assessment, he reported headache and cough but no other recent symptoms compatible with RVFV infection. He reported no direct contact with animals or recent travel outside of Kabale district.

### Additional acute case identification.

By March 27, 2016, after the initial outbreak investigations were concluded, three confirmed acute RVF cases (AC1, 2, and 3) had been identified, and an additional 19 suspected cases were investigated; all were negative for RVFV. Two months later on June 7, a 35-year-old male mason who resided in Rushaki village, Southern division, Kabale district, presented to KRRH with fever and symptom onset reported to be on June 2 and was admitted. Symptoms included vomiting, intense fatigue, abdominal pain, chest and muscle pains, headache, cough, conjunctivitis, sensitivity to light, and hemorrhage. A sample was collected on admission and sent to the UVRI for testing; it was found RVFV positive by RT-PCR (Ct = 21.3) on July 9 and confirmed as the 4 acute case 4 (AC4). The patient continued to receive supportive care at KRRH, and subsequent blood samples were drawn to monitor RVFV infection and serological response.

### Sequential molecular and serological testing.

**Figure 2.**
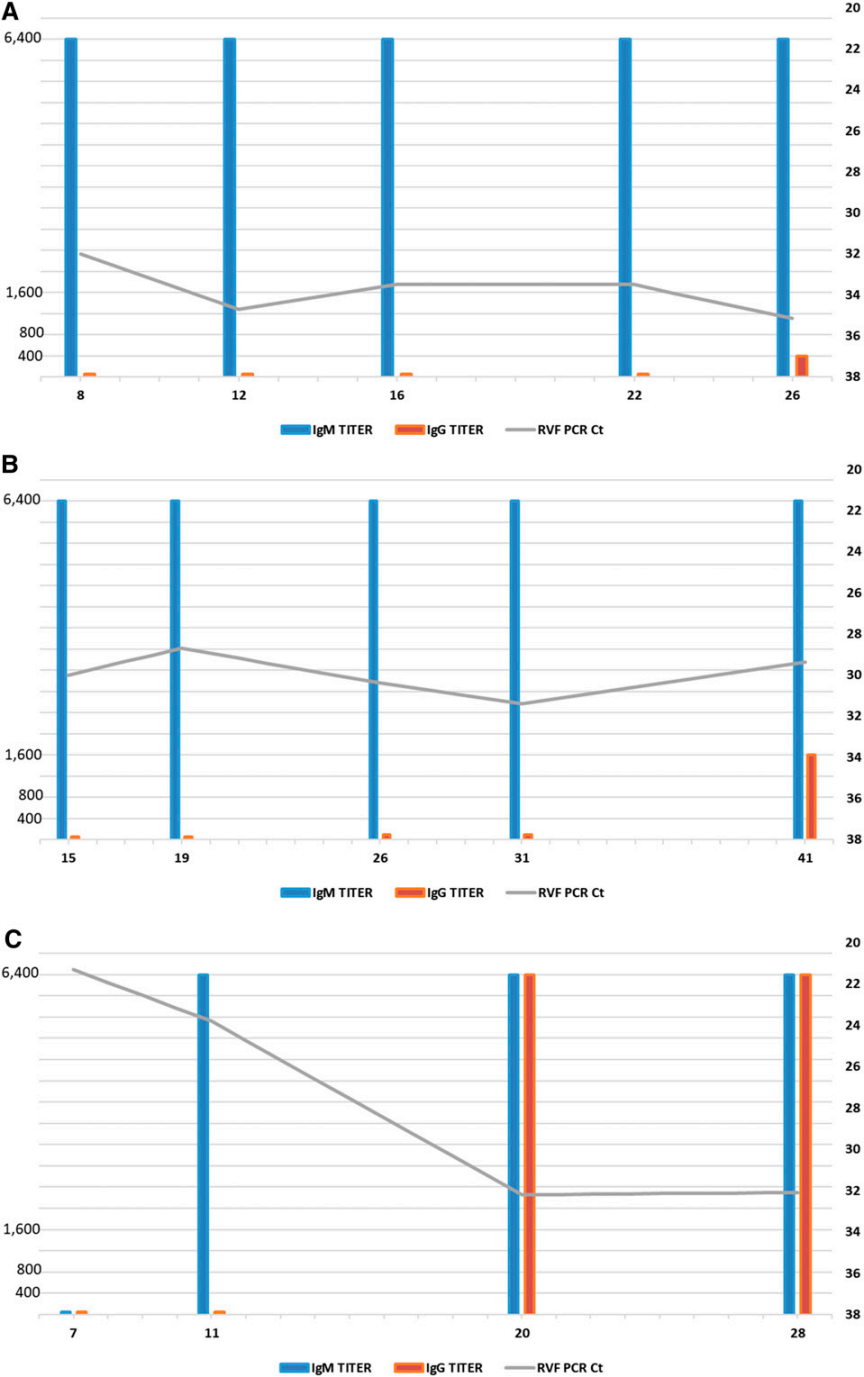
Sequential Rift Valley fever virus (RVFV) IgM and IgG serologies and reverse transcriptase polymerase chain reaction (RT-PCR) for the three confirmed cases in Kabale district for acute clinical RVF cases AC1 (**A**), AC2 (**B**), and AC3 (**C**). *X* axis represents days post RVF symptom onset when blood sample was collected; *y* axis (left) represents IgG serological titer; *y* axis (right) represents cycle threshold (Ct) values for RT-PCR performed on each sequential clinical sample. (**A**) RVFV sequential serology and RT-PCR for the initial confirmed acute case (AC1). (**B**) RVFV sequential serology and RT-PCR for the second confirmed acute case (AC2). (**C**) RVFV sequential serology and RT-PCR for the third confirmed acute case (AC3). This figure appears in color at www.ajtmh.org.

Following confirmation of acute RVFV in cases AC1 and 2, each was placed into isolation and provided supportive treatment. On March 9, following RVF confirmation, AC1 was transferred to Mbarara Regional Referral Hospital, approximately 140 km northeast of Kabale town, for isolation and treatment. This transfer was due to hemorrhagic manifestations and the heightened infection prevention and control practices requested because of the unfamiliarity in treating RVFV cases in Uganda. Mbarara hospital was the site of a large treatment and isolation center for Marburg hemorrhagic fever and also hosts a Médecins Sans Frontière epicenter office to provide additional medical support and guidance for treatment. Acute case 2 remained at KRRH for treatment. Successive blood samples were collected from each patient during treatment to monitor RVFV RNA levels in the blood by RT-PCR, and the progression of the patients’ immune response through IgM and IgG serology is shown in Figure 2.

On admission (8 days post symptom onset), AC1 had an RT-PCR Ct value of 32 and an IgM titer of ≥ 1:6,400, indicating acute infection. Subsequent blood samples were obtained over the course of the patient’s isolation 12, 16, 22, and 26 days post symptom onset. The viral Ct value first increased slightly (Ct = 34.7 on day 12) but remained relatively stable, with Ct values of 33.5, 33.5, and 35.15 on days 16, 22, and 26, respectively. IgM titers remained constant at ≥ 1:6,400 on all time points tested. IgG titer was undetectable throughout the course of isolation, except at day 26 post symptom onset, when IgG titer of 1:400 was found. No additional samples were taken and the patient was recommended for discharge on April 26, 2016.

Acute case 2 was admitted into isolation at KRRH 15 days after symptom onset, with an RT-PCR Ct value of 30 and an IgM titer of ≥ 1:6,400, indicating acute infection. Subsequent blood samples were taken 19, 26, 31, and 41 days post symptom onset. The viral Ct value decreased slightly at 19 days post onset to 28.7, increased on days 26 and 31 to 30.4 and 31.4, respectively, and then decreased slightly to 29.39 by day 41. As with the initial patient, IgM titers remained constant at ≥ 1:6,400, and no IgG was detected until day 41 (1:1,600 titer). No additional samples were taken and the patient was also recommended for discharge on April 26, 2016. He remained in isolation for supportive treatment after developing some ocular complications as a result of RVFV infection.

Acute case 3 was detected through the initial epidemiological investigations in the community and, thus, only one sample was collected on March 13.

Acute case 4 was admitted into isolation at KRRH on June 7, 7 days post symptom onset, with an initial RT-PCR Ct value of 21.3 and no detectible IgM or IgG titers. Subsequent blood draws were taken 11, 20, and 28 days post symptom onset. The viral Ct value increased slightly to 23.8 at 11 days post onset and then increased more dramatically to 32.2 and 32.1 on days 20 and 28, respectively. IgM was detectable on day 20, rising dramatically to ≥ 1:6,400 titer; this titer remained constant. IgG titer was first detected on day 20, with a ≥ 1:6,400 titer; this also remained constant. No additional samples were collected, and the patient was recommended for discharge.

### Livestock investigations.

In total, eight of 83 (9.6%) livestock were seropositive for RVFV by IgG ELISA (Table 1). Only one animal, a goat from Omururinda village where the second confirmed case resided, was also positive by RT-PCR (Ct = 33.3), showing acute infection. IgG serology confirmed the goat to have been infected (titer ≥ 1:6,400). An interview with the animal owner revealed that the goat had a previous abortion with the death of one kid in February 2016.

### Entomological investigations.

Results of the vector investigations are shown in Table 2. Overall, a total of 298 pools, representing 9,950 mosquitoes, were collected from the five locations listed previously. The predominate pooled species sorted for RT-PCR testing was *Aedes gibbinsi* (24.2%) followed by *Coquillettidia fuscopennata* (13.4%) and *Aedes tricholabis* (11.4%). In total, six genera and 33 species of mosquitoes were identified. Only three (1%) mosquito pools were found positive for RVFV by RT-PCR. One positive pool was *A. gibbinsi* (1.4%) trapped in Mushenyi village, home of the first probable case, PC1. The second positive pool was unspecified *Aedes* spp. (12.5%) trapped in Kazigizigi, Southern division, Kabale town. The third positive pool was *C. fuscopennata* (2.5%) trapped near the home of the second acute case, AC2.

**Table 2:** Summary of mosquito pools collected and tested by RT-PCR for Rift Valley fever virus

Subcounty	Bugongi	Kamuganguzi	Kazigizigi	Mushenyi	Nyakayenje			
Genus	Home of AC1	Home of AC2	Southern division, Kabale town	Home of PC1	Near Lake Bunyoni	Total	RT-PCR positive	% Positive
*Aedes*	3	59	40	52	1	155	–	–
*(Aedimorphus) dentatus*	–	–	6	–	–	6	–	–
*(Aedimorphus) gibbinsi*	2	33	7	30	–	72	1	1.39%
*(Aedimorphus) aegypti*	–	1	–	–	–	1	–	–
*(Aedimorphus) albothorax*	–	–	2	–	–	2	–	–
*(Aedimorphus) cumminsii*	–	6	6	2	–	14	–	–
*(Aedimorphus) domesticus*	–	6	–	–	–	6	–	–
*(Aedimorphus) phylolabis*	–	2	–	–	–	2	–	–
*(Aedimorphus) tarsalis*	–	–	2	–	–	2	–	–
*(Aedimorphus) tricholabis*	1	5	11	16	1	34	–	–
*(Finlaya) ingrami*	–	2	–	2	–	4	–	–
*(Neomelaniconion) circumluteolus*	–	1	1	2	–	4	–	–
*(Aedimorphus)* spp.	–	3	5	–	–	8	1	12.50%
Anopheles	–	5	1	2	–	8	–	–
*(Anopheles) coustani*	–	3	–	–	–	3	–	–
*(Anopheles) funestus*	–	–	–	1	–	1	–	–
*(Anopheles) implexus*	–	2	1	1	–	4	–	–
*Coquillettidia*	–	26	22	14	–	62	–	–
*(Coquillettidia) aurites*	–	3	1	1	–	5	–	–
*(Coquillettidia) fraseri*	–	–	1	–	–	1	–	–
*(Coquillettidia) fuscopennata*	–	15	14	11	–	40	1	2.50%
*(Coquillettidia) metallica*	–	3	6	1	–	10	–	–
*(Coquillettidia) pseudoconopas*	–	2	–	–	–	2	–	–
*(Coquillettidia) versicolor*	–	3	–	1	–	4	–	–
Culex	–	31	13	7	–	51	–	–
*(Culex) antennatus*	–	1	–	1	–	2	–	–
*(Culex) decens* group	–	8	8	6	–	22	–	–
*(Culex) neavei*	–	6	1	–	–	7	–	–
*(Culex) perfuscus*	–	3	–	–	–	3	–	–
*(Culex) rubinotus*	–	1	–	–	–	1	–	–
*(Culex) trifilatus*	–	2	2	–	–	4	–	–
*(Culex) univitattus*	–	3	–	–	–	3	–	–
*(Oculeomyia) annulioris*	–	3	1	–	–	4	–	–
*(Culex)* spp.	–	4	1	–	–	5	–	–
Mansonia	–	7	1	2	–	10	–	–
*(Mansonioides) nigerrima*	–	5	1	2	–	8	–	–
*(Mansonioides) uniformis*	–	2	–	–	–	2	–	–
Misc.	–	4	1	–	–	5	–	–
*(Culiciomyia) nebulosus*	–	1	–	–	–	1	–	–
*(Metalutzia) tigripes*	–	2	1	–	–	3	–	–
*(Stegomyia) simpsoni*	–	1	–	–	–	1	–	–
Unknown	–	–	7	–	–	7	–	–
spp. (no legs)	–	–	7	–	–	7	–	–
Grand total	3	132	85	77	1	298	3	1.01%

RT-PCR = reverse transcriptase polymerase chain reaction.

### Virus isolation.

Virus isolation was attempted using all blood samples serially collected from the three RT-PCR–positive acute RVF cases, AC1, 2, and 4. Rift Valley fever virus was only able to be isolated from the first acute sample drawn from each patient (Figure 2). Subsequent samples did not yield any virus isolates in cell culture. The viral isolates obtained from these initial samples were used for genetic sequencing.

### Genetic sequencing of RVFV from human samples.

**Figure 3.**
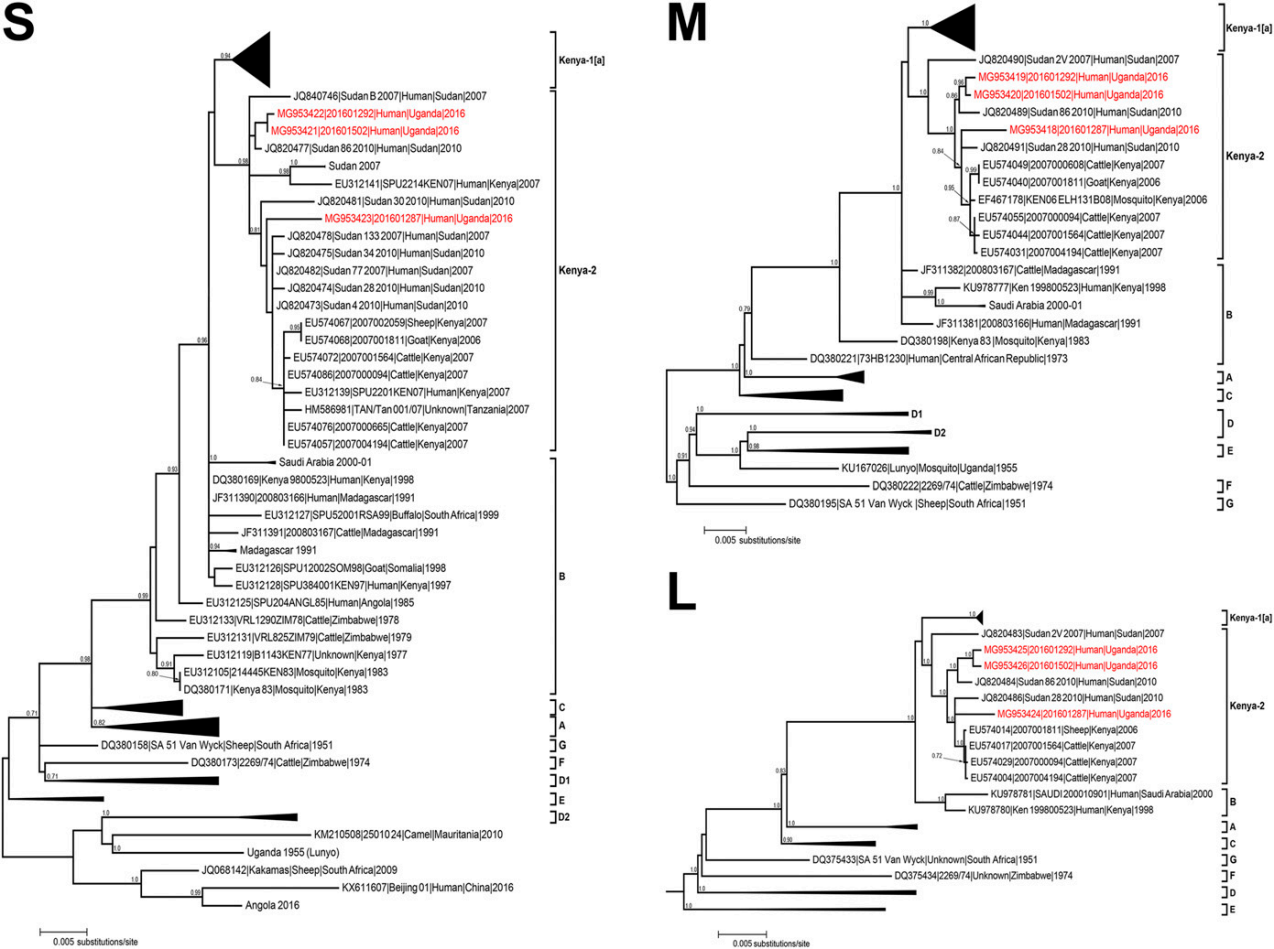
Phylogenetic trees comparing complete S (A), M (B), and L (C) segment sequences of RVFV using all available full genome sequences. The sequence from the three RT-PCR–positive acute human cases described here, 20160187 (AC1), 201601298 (AC2), and 201601502 (AC4), are in red type. The evolutionary history was inferred based on the SPR model with the GTR + Γ (*n* = 4) nucleotide substitution model. The tree is drawn to scale, with branch lengths measured in the number of substitutions per site. Branch support estimates represent bootstrap values following 1,000 replicates and are displayed as integers for branch support > 70%. Clades are labeled according to Bird et al.^24, 26^ and Aradaib et al.^27^ Evolutionary analyses were conducted using phyml (v 3.0). Scale bar represents 0.005 substitutions/site. GenBank accession numbers used in tree are MG953418, MG953419, MG953420, MG953421, MG953422, MG953423, MG953424, MG953425, and MG953426. RT-PCR = reverse transcriptase polymerase chain reaction; RVFV = Rift Valley fever virus. This figure appears in color at www.ajtmh.org.

We were able to obtain complete S, M, and L segment genome sequences from cases AC1, 2, and 4. All three complete RVFV RNA segment genome data were obtained directly from the first clinical samples sent to UVRI for diagnostic testing and subsequently sent to CDC in Atlanta, GA, for further processing and analysis. All three segments showed the closest genetic relationship to previously identified RVFV isolated in Kenya in 2007 and Sudan in 2010 (Figure 3). There was little difference in the overall nucleotide lengths of the S, M, and L segments of the three Kabale isolates compared with the Kenya-2007 and Sudan-2010 sequences: S segment was 1,690–1,691 nt, M segment was 3,885 nt, and L segment was 6,404 nt. Pairwise nucleotide identity between the three Kabale RVFV isolates ranged from 94.7% to 98.4% for S segment, 99.3–99.8% for M segment, and 99.2–99.9% for L segment (Supplemental Table 1). Amino acid identity between the three isolates was 100% for S segment, 99.2–100% for M segment, and 99.8–99.9% for L segment (Supplemental Table 2).

Maximum likelihood phylogenetic analyses of the three Kabale RVFV isolates showed them all clustering in the same positions regardless of the segment analyzed. Sequences from the first acute Kabale case identified (201601287) were grouped in the Kenya-2 clade. All sequences were closely related to human sequences identified in Sudan in 2007 and 2010, and in Kenya in 2007. Viruses within the Kenya-2 clade have been previously associated with the large epizootic occurring in East Africa in 2006–2007,^24^ whereas the Sudan-2010 lineage was associated with human cases identified as part of enhanced surveillance efforts in Sudan following an epizootic that occurred in several Sudanese states in 2006–2007 and was linked with the larger East African epizootic during the same time period.^28^ No genetic evidence of multiple RVFV lineages circulating in Kabale district was seen nor was any evidence of virus reassortment within the samples analyzed. Even though these sequences were obtained from geographically distinct locations within Kabale district, sequence analysis shows that these isolates belong to a singular lineage and suggests at least two introductions into Kabale district. These sequence data suggest that the progenitor viruses of the 2016 Kabale RVFV outbreak likely belonged to Kenya-2 clade, which was also responsible for the outbreak in East Africa in 2006–2007.

## DISCUSSION

This outbreak of RVFV in Kabale district represents the first confirmed human cases of RVF in Uganda since 1968. In total, four laboratory-confirmed acute cases (AC1-4) and two probable deaths (PC1 and 2) were identified. The three acute RT-PCR positive cases (AC1, 2, and 4) were identified at health-care facilities, with one IgM-positive acute case (AC3) identified through laboratory testing of community members residing in affected villages. A multidisciplinary team carried out a rapid and comprehensive outbreak investigation within 2 days of initial laboratory confirmation, including collection of human, livestock, and vector samples to identify additional cases and possible sources of infection. Laboratory samples collected from 19 family and community members of confirmed and probable cases revealed two additional seropositive, convalescent RVF cases (CC1 and CC2), showing that RVFV has been circulating in Kabale district before detection of this outbreak.

A total of 83 livestock samples were collected, with 8 (9.6%) positive by IgG serology, showing evidence of previous infection with RVFV. IgM serology was not performed to determine how many livestock were recently infected, but we did perform RT-PCR testing on all livestock to determine if any were actively infected. One caprine sample from the home village of AC1 was found to be RVFV positive by RT-PCR, showing acute infection and suggesting active RVFV transmission was occurring at the time of the investigation. In addition, a total of six different genera of mosquito vectors, representing 33 species/subspecies, were collected near the locations of the confirmed and probable cases. Rift Valley fever virus was detected in three of 298 (1%) pools tested by RT-PCR, confirming RVFV-infected vectors and suggesting that mosquito-borne virus transmission was also occurring at the time of the outbreak.

Multiple RVF epidemics have been documented in Uganda, the last occurring in 1968 and involving seven individuals living in the Entebbe area, near EAVRI (now UVRI).^15^ These individuals lived near a forested area on the outskirts of Entebbe and presented to the EAVRI clinic within 1–5 days of onset of fever, headache, abdominal pain, vomiting, and chills. These symptoms are very similar to the cases presenting from this recent outbreak in Kabale. Because only seven confirmed human cases were documented in the Entebbe outbreak, RVFV transmission was probably limited, indicating maintenance transmission or incidental transmission from enzootic maintenance rather than a widespread epizootic.

An entomological investigation was initiated during this most recent outbreak, with evidence of acute infection in one caprine. The presence of RVFV in Uganda has previously been confirmed on multiple occasions in humans, livestock, and mosquitoes. Most notably, the virus had been detected in *Aedes africanus* and *Aedes circumluteolus* mosquitoes in 1955 in Lunyo near Entebbe; in 1960 from febrile patients near EAVRI, along with isolations from *Mansonia africana* and *Mansonia uniformis*; and again in 1963 from two febrile patients from the Entebbe area.^15,29,30^

Rift Valley fever virus is thought to be endemic in the indigenous forest areas of East Africa. The virus is thought to circulate in *Eretmapodites* spp. mosquitoes and unknown vertebrates in the forests. It spreads in seasons of exceptionally heavy rainfall to livestock-rearing areas, where flood water–breeding aedine mosquitoes of the *Aedimorphus* and *Neomelaniconion* subgenera may be transovarially infected and then transmit the virus to animals and, less commonly, to humans.^31^ However, other mosquito species may serve as RVFV vectors as well. Earlier studies by Smithburn et al.,^10^ Weinbren et al.,^32^ and Williams et al.^11^ indicated that RVFV occurs naturally in a number of mosquito species providing a mechanism for maintenance during enzootic periods and include *A. africanus*, *Aedes (Stegomyia) dendrophilus*, *Aedes (Stegomyia) aegypti*, *Aedes (Aedimorphus) argenteopunctatus*, *Aedes (Aedimorphus) tarsalis*, *A. circumluteolus*, *C. fuscopennata*, *Coquilletidia pertubans*, and *Eretmapodites* spp.^10,11,32^

In our survey, *A. gibbinsi* was the most abundant mosquito species collected. *Aedes gibbinsi* has been described as a potential epizootic, as well as a possible reservoir vector, of RVFV.^33^ It is a probable enzootic maintenance vector of the virus in Zimbabwe and may have played a similar role in this outbreak. It is a zoophilic mosquito species that likely does not transmit the virus directly to humans. *Aedes tricholabis* is also a zoophilic species. Although humans primarily become infected with RVFV from contact with infected animals or tissues, species such as *C. fuscopennata* and *Mansonia* spp. may also bite and transmit RVFV to humans.

One pool of *A. gibbinsi* and one pool of *C. fuscopennata* mosquitoes were found to be RVFV positive by RT-PCR. *Aedes gibbinsi* mosquitoes have never been implicated in RVFV transmission, although other members of the *Aedimorphus* subgenera, such as *Aedes argenteopunctatus and Aedes tarsalis*, are known RVFV vectors; thus, *A. gibbinsi* may also be involved in RVFV enzootic maintenance transmission. *Coquillettidia fuscopennata* were the most abundant mosquito collected, and are known RVFV vectors. This species was implicated in the 1960 RVFV outbreak in Entebbe.^11^ However, only one pool of these mosquitoes was RVFV positive, suggesting that these arthropods may not be the major vector responsible for causing acute human cases in this current outbreak. Alternatively, these mosquitoes may have been collected after the peak of the outbreak, when relatively few mosquitoes remained infected with RVFV. Additional vector surveillance and testing should be carried out in Kabale to monitor vector infectivity and potential transmission.

In contrast to epizootics in Kenya and Tanzania, which involved more than 300 cases each, only four acute RVFV cases were identified in Kabale between March and July of 2016.^8,9^ These identified cases likely represent more severe and complicated RVFV infections and were identified because the patients sought care. Likely, the total number of infected persons in the region was much higher. Our investigations revealed a low level of seropositivity, with only two of 19 samples containing detectable levels of IgG and only one of the four acute cases having both IgM and IgG. No additional RT-PCR–positive human cases were detected, indicating that this outbreak was not part of a large, expansive epizootic like the ones that occurred in neighboring countries. For comparison, overall human IgG seropositivity was 0.7–23% in Kenya, with a maximum local seroprevalence of 29%, and 5.2–11.7% in Tanzania, with a maximum local seroprevalence of 29.3%.^34–39^

The limited nature of the epizootic in Kabale district may be due in part to the district’s geography. The region has many isolated hills and valleys, creating pockets of enzootic activity and isolated virus maintenance between mosquitoes and livestock. Humans may be only incidentally infected, either directly by mosquito vectors or by exposure to infected livestock. Another reason for the limited number of human RVFV cases may be the lack of vectors that both transmit RVFV and preferentially feed on humans.

During our initial investigations, we sampled livestock in the villages and homes of confirmed and probable cases for evidence of previous or current RVFV infection. Overall, 10% of livestock sampled were seropositive for RVFV, with one caprine also positive for RVFV by RT-PCR. These results suggest that RVFV transmission has been occurring in Kabale district, although there were no reported increases in the number of animals with signs of RVFV infection or in overall animal morbidity or mortality from the locations where animal samples were collected. Only two of the animals were reported to have had an abortion in the months before sampling. These findings also suggest there may not have been widespread RVFV transmission occurring in Kabale district at the time of the detected human infections. The results from our human and livestock serological analysis are similar to what has been reported in human and domestic livestock populations during interepidemic periods in Kenya and Tanzania.^40–42^

We would expect to have seen a much higher percentage of seropositive livestock if the emergence of RVFV in Kabale was similar to that in Kenya and Tanzania. Previous studies in East Africa have shown livestock seropositivity to be between 13.1% and 27.6% in Kenya and 8.2% from a single study in Tanzania.^40–42^ To more accurately characterize the epizootiology of RVFV in Kabale and neighboring districts, UVRI in collaboration with MAAIF, Uganda MOH, and Kabale district officials conducted a comprehensive serosurvey in humans and livestock. The results of this survey will be reported separately and should provide valuable information on the endemicity and spread of RVFV in the southwestern region of Uganda.

We identified seropositive or acutely positive animals in all locations where a human case was either confirmed or suspected. Although 10% of animals tested were RVFV seropositive, these data, at minimum, show evidence of RVFV circulation. The identification of one RT-PCR–positive caprine shows evidence of active circulation of RVFV in at least one location associated with acute human cases. Our investigations were limited in size and scope because of focusing on identifying additional cases and possible sources of infection related to the first identified acute cases; a larger serosurvey will be required to more accurately characterize enzootic transmission and RVFV endemicity in Uganda.

We were able to obtain serial clinical samples from three of the four patients with acute, RT-PCR–positive, nonfatal RVF infection, allowing comprehensive laboratory monitoring of the antibody response and RNA viral loads in these individuals. Serum IgM titers were initially high (≥ 1:6,400) in the first two patients (AC1 and 2), suggesting a robust early immune response, although these samples were collected eight and 15 days post symptom onset. IgG titers, on the other hand, were undetectable until the final sample collection on days 26 and 41. Reverse transcriptase polymerase chain reaction Ct values remained steady throughout the time course of infection despite evidence of a mounting immune response shown by rising antibody levels. No IgM was detected in the initial sample from the fourth acute case (AC4), but rising IgM and IgG titers were seen in the subsequent samples. Reverse transcriptase polymerase chain reaction Ct values decreased in this patient over time, indicating clearing of virus.

These results are as expected for nonfatal RVFV infections and are similar to previously studied serially collected samples tested during an outbreak in Saudi Arabia.^5^ The primary difference we see is the consistent Ct values throughout the course of infection cases AC one and two despite an immune response, suggesting some maintenance of RNAemia despite an active immune response. No fatal cases were studied in Kabale district, unlike in Saudi Arabia; fatal cases in that outbreak corresponded to very high initial Ct values that remained high throughout monitoring with no detectable immune response, which is indicative of fatal outcomes.^5^ The fourth acute case (AC4) studied initially had high Ct values that steadily declined over time. We were only able to isolate live RVFV from the first serially collected RT-PCR–positive Kabale patient samples, indicating that the detectable RNA in subsequent samples may not equate to infectious virus but rather RNA lingering in serum within immune complexes or similar modifications. It is also possible that the prior handling, transport, and freezing and thawing of these clinical samples may have negatively affected the sensitive viral isolation assay and, thus, we cannot definitively state that no infectious virus remains after 8 days of symptom onset.

Rift Valley fever virus and antibody levels were measured to assist medical staff monitoring of patients’ clinical courses in addition to their immunologic responses and to determine discharge criteria. The viral and serological dynamics seen in these serially collected samples again show that samples collected at regular intervals may be prognostic for clinical outcome. Our results indicate that RNAemia can remain detectable up to 41 days post symptom onset, potentially complicating classification of truly acute cases. Performing IgM and IgG ELISA in addition to RT-PCR is vitally important in providing a complete profile of RVFV infection that may not follow a typical time course.

The three viral isolates sequenced in our study are all most closely related to viral lineages from previous RVFV epizootics in Kenya and Tanzania in 2007 and in Sudan in 2010. Previous studies examining the ancestry and genetic relationships of the viruses isolated during the 1997–1998 and 2006–2007 outbreaks in Kenya show that a single RVFV lineage can reemerge after long periods of latency to again cause widespread epizootics.^24^ Our analysis shows that all three segments (S, M, and L) of the Kabale-2016 viruses are most closely related to viruses from the Kenya-2 clade, identified during the last epizootic in 2006–2007 and a direct descendant of the 1997–1998 epizootic RVFV. This demonstrates ongoing viral activity and maintenance of these lineages during interepidemic periods throughout East Africa. The Kabale isolates are also related to RVFV isolated in Sudan in 2007 and 2010. The 2007 Sudan isolates were obtained during the larger East Africa epizootic/epidemic occurring at the time, whereas the 2010 isolates were a more limited emergence in El Gezira state, south of Khartoum.^27^ Both of these Sudan isolates fall within the Kenya-2 clade. Bayesian analysis of sequence differences between the 2007 and 2010 Sudan RVFV variants, as well as the 2006–2007 Kenyan variants, show all these viruses share a most recent common ancestor from the 1997–1998 East African epizootic.^24,27^ These findings all reaffirm the maintenance of viruses of this lineage in East Africa over long periods of time.

How or when RVFV was introduced into Kabale district or southwestern Uganda is unknown. One study conducted in 2009, shortly after the 2006–2007 East African epizootic, examined IgG prevalence in goat populations in the neighboring districts of Sembabule, Mpigi, Masaka, and Mubende. The study showed overall IgG seroprevalence of 9.8% (144/1,470), a rate consistent with what we found during our livestock investigations, which could indicate environmental endemic maintenance of RVFV following the large epizootic in Kenya and Tanzania. Although the testing methodology may have overestimated seroprevalence, it nonetheless provides evidence of possible RVFV circulation in livestock for at least 7 years before the first human cases were detected in Kabale district in 2016 and indicates that RVFV was introduced into the southwestern Uganda region in 2006 or shortly after. However, previous livestock seroprevalence studies conducted by the UVRI/CDC VHF program in other districts in central, northern, and eastern Uganda between 2011 and 2016 showed no serological evidence of RVFV.

Another factor contributing to the emergence of RVFV in Kabale district could be the influence of El Niño weather events. In 2015, the National Oceanic and Atmospheric Administration issued an emerging health risk notification for an increased risk of RVFV outbreaks.^43^ The 2015 El Niño weather pattern was estimated to be one of the top three events recorded resulting in substantially increased rainfall in Sudan, South Sudan, Ethiopia, Somalia, Kenya, and Tanzania, peaking in late 2015 and early 2016. The cases identified in Kabale district may have been a result of the El Niño event influencing environmental factors, thereby increasing vector populations and the probability of spillover to humans. The southern part of Uganda normally has two rainy seasons: one from mid-September to November and another from March until May (Supplemental Figure 1). Meteorological data obtained from the Uganda national meteorological service show that in late 2015, the rainy period in Kabale district began in September but lasted into January 2016, 2 months longer than normal (the second rainy period occurred as expected). Moreover, total rainfall (241.6 mm) between January and March 2016 was noticeably higher than the total during the same time period of the previous year (166.3 mm). The total rainfall in January 2016 was 85.8 mm, compared with 9.8 mm in 2015 and 1.8 mm for 2014. In March 2016, when the first RVF cases were identified, the rainfall was 124.7 mm compared with 32 mm in 2015. This earlier, heavier rainy season in 2016 than in 2015 may have contributed to increases in competent mosquito vector populations. We suspect RVFV to have been circulating in Kabale district for years, but rainfall and other weather conditions in 2016 may have allowed its transition to an epizootic. Additional analyses of weather and other ecological factors contributing to emergence of RVFV in Kabale are planned.

National Aeronautics and Space Administration (NASA), United States Department of Agriculture (USDA), and the United States Department of Defense (DoD) developed an RVFV forecasting model that uses satellite-derived data and is based on the 2006–2007 El Niño event that led to the large RVFV epizootic in East Africa at that time.^44^ A risk map highlights portions of northern Rwanda and Uganda, including areas of Kabale district, as “potential epizootic areas (areas with previous known or predicted presence of RVF virus).” The model also identified large areas of Tanzania, Kenya, Somalia, Ethiopia, and southern Sudan as “potential epizootic areas with recent heavy rainfall,” or areas more likely to have an RVFV outbreak. Although to our knowledge no large-scale outbreaks have been reported in these potential high-risk areas at the time, the emerging health risk notifications are issued to allow countries to begin implementation of effective control measures to mitigate a potential RVFV outbreak. These measures include enhancing surveillance, vaccinating livestock, vector control activities, and risk communication. Currently, no routine vector control or RVFV vaccination activities are carried out in Uganda, so advanced notice for beginning these activities could be greatly beneficial.

Beginning in 2011, the Viral Special Pathogens Branch of CDC-Uganda, in collaboration with UVRI and the Uganda MOH, established a first of its kind national VHF surveillance and laboratory program. The program allowed the identification of several VHF outbreaks, including a Marburg virus disease outbreak in Kabale district in 2012. The 2016 RVFV outbreak represents the 10^th^ independent VHF outbreak detected and confirmed through this program. Viral hemorrhagic fever outbreaks tend to become high profile and generate widespread media attention, including nationwide health messaging and awareness, and so most districts have become sensitized to cases of severe disease that could be suspected VHFs. The surveillance system was enhanced in 2013 with the expansion of the national sample transportation network and establishment of the Public Health Operations Center funded through the Global Health Security Agenda to rapidly transport suspected clinical specimens from regional hubs to the national reference laboratories.^45^ This network includes UVRI, which serves as the national reference laboratory for VHFs in Uganda, and allowed the rapid detection, laboratory confirmation, and outbreak response to this first outbreak of RVFV in Kabale. This rapid response demonstrates the significant public health impact of having established, ongoing, dedicated VHF surveillance to mitigate morbidity and mortality of endemic zoonotic infectious diseases.

## Supplementary Files

Supplemental tables and figure
