# Supplementary material for: First Laboratory-Confirmed Outbreak of Human and Animal Rift Valley Fever Virus in Uganda in 48 Years
Source: Am J Trop Med Hyg. 2019 Jan 21;100(3):659–71. doi: 10.4269/ajtmh.18-0732 (PMC6402942; doi:10.4269/ajtmh.18-0732)
Supplement: Supplementary file 1 [file tpmd180732.SD1.pdf]

The following are supplemental materials and will be published online only

Supplemental Figures

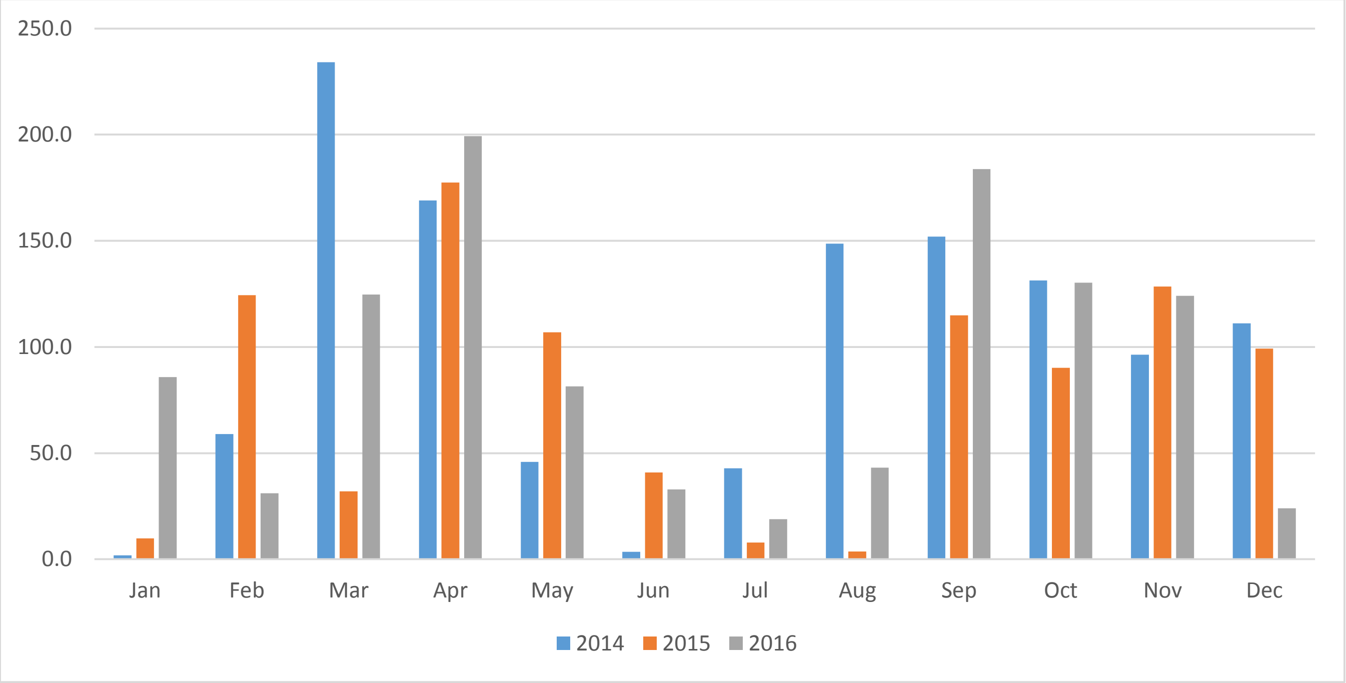

Figure S1. Monthly cumulative rainfall totals (mm) for Kabale district by month, 2014-2016.

Supplemental Tables

		# Nucleotide Differences		
	% Coverage	201601287	201601292	201601502
S	201601287	94.558 (93)	75	83
	201601292	94.709	98.069 (33)	10
	201601502	94.771	98.339	93.078 (37)
M	201601287	90.018 (396)	27	26
	201601292	99.245	97.202 (111)	6
	201601502	99.273	99.843	96.547 (137)
L	201601287	96.229 (244)	50	50
	201601292	99.193	98.490 (98)	9
	201601502	99.193	99.857	98.377 (105)
	Nucleotide % Identity			

Table S1. Pairwise percent identity matrix comparison of nucleotides in the of 3 whole genome sequences from clinical samples obtained during the 2016 RVF outbreak in Kabale district, Uganda. L, M, and S indicate genomic segments of RVFV. Gray-shaded cells indicate the percent coverage of the respective genomes with the total number of missing nucleotides (in parentheses). In most instances, missing nucleotides occurred on the terminal ends of the sequence with the exception of the S segment, where each sample was missing approximately 26–95 bps in the 828–924 nucleotide non-coding region. The cells below the gray cells present the pairwise percent nucleotide identity, and the cells above present the total number of nucleotide differences for each RNA segment.

		# Amino Acid Differences		
	% Coverage	201601287	201601292	201601502
NP (S)	201601287	98.836 (3)	0	0
	201601292	100	100	0
	201601502	100	100	100
NSs (S)	201601287	97.444 (5)	2	2
	201601292	99.231	100	0
	201601502	99.231	100	100
NSm, Gn, Gc (M)	201601287	98.663 (16)	5	3
	201601292	99.577	100	2
	201601502	99.746	99.832	99.415 (7)
Polymerase (L)	201601287	98.614 (29)	5	5
	201601292	99.758	100	2
	201601502	99.758	99.904	99.904 (2)
		Amino Acid % Similarity		

Table S2. Amino acid similarity of gene products of the 3 whole genome sequences from clinical samples obtained during the 2016 RVF outbreak in Kabale district, Uganda. Encoded proteins are given with the segment on which they are encoded provided in parentheses. Gray-shaded cells indicate the percent coverage of the genomes, with the total number of missing amino acids (in parentheses). In all instances, missing amino acids were at the terminal translation sites as a result of missing nucleotides.

The cells below the gray present the pairwise percent amino acid similarity, and the cells above present the total number of amino acid differences for each RNA segment.
